# Among respiratory symptoms, wheeze associates most strongly with impaired lung function in adults with asthma: a long-term prospective cohort study

**DOI:** 10.1136/bmjresp-2021-000981

**Published:** 2021-07-18

**Authors:** Nicolás Bermúdez Barón, Anne Lindberg, Caroline Stridsman, Martin Andersson, Linnea Hedman, Sigrid Anna Vikjord, Hannu Kankaanranta, Bo Lundbäck, Eva Rönmark, Helena Backman

**Affiliations:** 1 Department of Public Health and Clinical Medicine, Section of Sustainable Health, The OLIN Unit, Umeå University, Umeå, Sweden; 2 Department of Public Health and Clinical Medicine, Section of Medicine, The OLIN Unit, Umeå University, Umeå, Sweden; 3 Department of Health Sciences, Division of Nursing, Luleå University of Technology, Luleå, Sweden; 4 HUNT Research Centre, Department of Public Health and Nursing, Faculty of Medicine and Health Sciences, Norwegian University of Science and Technology, Trondheim, Norway; 5 Department of Respiratory Medicine, Seinäjoki Central Hospital, Seinäjoki, Finland; 6 Faculty of Medicine and Health Technology, Tampere University, Tampere, Finland; 7 Department of Internal Medicine and Clinical Nutrition, Krefting Research Centre, University of Gothenburg Institute of Medicine, Goteborg, Sweden

**Keywords:** asthma epidemiology, clinical epidemiology

## Abstract

**Background:**

Asthma is a common disease and a major public health concern. Respiratory symptoms are related to its prognosis, which in turn associates with lung function. Still this association on a long-term basis is not entirely understood.

**Aim:**

To study the association of the type and number of respiratory symptoms with FEV_1_ and FEV_1_ decline in women and men with asthma.

**Method:**

A population-based cohort of adults with asthma was examined at study entry between 1986 and 2001 and at follow-up between 2012 and 2014, and n=977 had valid measurements of FEV_1_ on both occasions. Data regarding respiratory symptoms at study entry (recurrent wheeze, dyspnoea, longstanding cough and productive cough) were analysed in relation to FEV_1_ and annual decline in FEV_1_, both unadjusted and adjusted for other potentially associated factors by linear regression.

**Results:**

For both sexes recurrent wheeze and dyspnoea were associated with lower FEV_1_ at study entry and follow-up, while productive cough was associated with lower FEV_1_ only at follow-up. No associations were found between the type of symptoms and annual decline in FEV_1_. In adjusted analyses, the association between recurrent wheeze and lower FEV_1_ both at study entry and follow-up remained significant among women. Also, the association between a higher number of symptoms with lower FEV_1_ both at study entry and follow-up were present for both sexes and remained after adjustment.

**Conclusions:**

Particularly recurrent wheeze and a higher number of respiratory symptoms may predict lower lung function also in the long run among women and men with asthma.

**Key messages**

- How does the type/number of respiratory symptoms associate with lung function decline on a long-term basis among women and men with asthma?
- Among respiratory symptoms, wheeze associates most strongly with impaired lung function in women and men with asthma. For both sexes the greater number of respiratory symptoms, the lower forced expiratory volume in 1 s values over time.
- Long-term population-based studies on lung function and respiratory symptoms among adults with asthma are scarce. This study gives new knowledge opening possibilities to future studies focusing on this field. The results may be used to assess a more accurate prognosis of asthma depending on the symptom presentation.

## Introduction

Asthma is a common respiratory disease and a major public health concern associated with impaired quality of life and an increased risk of both morbidity and mortality.^1–3^ It is characterised by chronic airway inflammation, variable airflow limitation and by respiratory symptoms such as wheeze, shortness of breath, chest tightness, cough, and mucosal hypersecretion; with variable presentations and intensity.^4^ Asthma is known to be associated with allergy, sex, body mass index (BMI), smoking, adherence to treatment, family history, lifestyle, socioeconomic status and other environmental factors.^5 6^ Although a heterogeneous disease and today known to have different phenotypes,^4^ it is often divided into allergic and non-allergic asthma.

Both respiratory symptoms^7–9^ and lung function decline^9–11^ are individually associated with higher mortality rates. Poor symptom control is one of the most common reasons for adults with asthma to visit the emergency department and seek medical attention.^12^ In addition, decline in lung function, represented by the forced expiratory volume in 1 s (FEV_1_), is more accelerated among adults with asthma than adults without asthma.^13^ Associations between respiratory symptoms and impaired lung function have been studied before, where cough may be of particular importance.^14–16^ Still, large prospective population-based studies among adults with asthma addressing the relation between respiratory symptoms and lung function decline are lacking and it is unclear if some symptoms may have stronger associations with lung function decline than others.

Thus, our aim was to establish how the type and number of respiratory symptoms at study entry were associated with FEV_1_ as well as changes in FEV_1_ in a prospective cohort study including women and men with asthma.

## Materials and methods

### The OLIN adult asthma cohort

#### Study entry

The obstructive lung disease in northern Sweden (OLIN) studies is an epidemiological research programme ongoing since 1985. Within OLIN, a population-based cohort of adults with asthma living in the northernmost county of Sweden, Norrbotten, (n=2055, 55% women, aged 19–72 years) was identified from clinical examinations of five original population-based cohorts performed between 1986 and 2001 (I in 1986–1987, II in 1993–1994, III in 1994–1995, IV in 1997–2001 and V in 1995–1999). Original cohorts I–IV were sampled from the population registry,^17^ while original cohort V was composed exclusively by adults with recent asthma onset, yet without treatment.^18^ Strict predefined criteria of asthma were used in all cohorts, and subjects with physician-diagnosed asthma and subjects with asthma identified at the clinical examinations, but not by healthcare prior to the examinations, were included in this asthma cohort. The examination included spirometry and a detailed structured interview about respiratory symptoms, diagnosis and treatment of obstructive respiratory diseases, as well as demographics, potential risk factors and occupation.^17^

#### Follow-up

From study entry until the follow-up in 2012–2014, 466 subjects were deceased and 164 had moved from the county, while the remaining 1425 were invited to the follow-up. In total, 1006 (71% of invited) participated in spirometry and detailed structured interviews. The mean follow-up time was 18 years (min-max 10–28 years).^17^

Subjects with valid lung function measurements both at study entry and at follow-up were included in the present study (n=977).

### Patient and public involvement

Beside what is described above regarding involvement in study conduct, a selection of participants was involved in pilot studies at the beginning of the follow-up where they advised on the included questions and order of the included clinical examinations. All participants will be informed of the results through a dedicated website (www.norrbotten.se/olin).

### Spirometry at study entry and follow-up

At study entry, spirometry was performed with a Mijnhardt Vicatest 5 dry volume spirometer with a repeatability criterion of the two best FEV_1_ measurements of ≤5%, and of ≤100 mL when the best FEV_1_ and forced vital capacity (FVC), respectively, was ≤1 L.^19 20^

At follow-up, spirometry was performed according to the 2005 ERS/ATS guidelines with a Jaeger Masterscope pneumotach spirometer with a repeatability criterion of ≤150 mL.^21^

FEV_1_ before bronchodilatation (pre-BD) was used, expressed as percent of predicted (FEV_1_pp) using the OLIN reference values.^22^ The annual decline in FEV_1_ was calculated as the value at follow-up minus the value at study entry divided by the exact time interval in between, expressed in absolute terms of FEV_1_ in pp.

### Definitions

#### Respiratory symptoms at study entry

Symptoms were assessed using a questionnaire developed for the OLIN studies^23^ which has been used in several studies in northern Europe and externally validated against the GA2LEN questionnaire and described in detail elsewhere.^24^ Internal missing on symptom questions were regarded as non-affirmative and included in the reference category “No/don’t know”.

*Recurrent wheeze* was defined by a positive answer to “Do you usually have wheezing or whistling in your chest when breathing?”.

*Dyspnoea* was defined as score ≥2 on the modified Medical Research Council dyspnoea scale (ranging from 0 to 4) where two corresponded to “walks slower than people of the same age because of dyspnea or has to stop for breath when walking at own pace”.

*Longstanding cough* was defined as answering yes to “Have you had longstanding cough during the last years?”.

*Productive cough* was defined as bringing up phlegm when coughing on most days during periods of at least 3 months.

Four categories were defined depending on the number of symptoms listed above: 0–1, 2, 3 and 4 symptoms.

#### Other definitions

##### At study entry

Data at study entry included sex, age, asthma medication use in the last 12 months (inhaled corticosteroids (ICS) or any asthma medication), BMI and smoking habits. BMI groups were categorised as underweight (BMI <20), normal (20≤BMI<25), overweight (25≤BMI<30), obese (BMI ≥30) and BMI missing. Smoking habits were categorised as non-smoker, ex-smoker (stopped smoking more than 1 year ago) and current smoker. Allergic rhinitis was defined by affirmative answer both to the question “Are you often bothered by nasal congestion or runny nose?” and reporting hay fever.

##### Changes between study entry and follow-up

Change in BMI was calculated as BMI at follow-up minus BMI at study entry and those with missing BMI (n=26) were excluded. A BMI cut-off point at the third quartile of the study sample was used to categorise subjects with high BMI increase (>4.92 increase).

Changes in smoking habits from study entry to follow-up were categorised as: never smokers (non-smokers at both occasions), ex-smokers (from non-smokers or ex-smokers to ex-smokers), quitters (from current smokers to ex-smokers), current smokers (from non-smokers or ex-smokers or current smokers to current smokers) and inconsistent (ex-smoker to non-smoker and current smoker to non-smoker).

##### At follow-up

Follow-up data included ICS use in the last 12 months, as well as occupational exposure to gas, dust or fumes (GDF) which was defined as answering yes to the question “Have you been heavily exposed to dust, gases or fumes at your work (not including tobacco)?”. Data on occupational exposure to GDF were not available at study entry.

### Statistical analyses

The analyses were made with the IBM SPSS Statistics V.25. Comparisons of means between two groups were performed by t-test and across more than two groups by analysis of variance (ANOVA). P<0.05 from two-sided tests were considered statistically significant.

Due to the long follow-up time, a substantial proportion were deceased and did not participate at follow-up. To evaluate potential selection bias, comparisons of mean FEV_1_ between those with and without individual respiratory symptoms were also performed separately for the 1052 subjects with valid spirometry data at study entry that did not participate at follow-up.

For the cross-sectional analysis of the association between FEV_1_ and respiratory symptoms at study entry, a linear regression model was constructed including recurrent wheeze, dyspnoea, longstanding cough, productive cough, age, BMI category and smoking habits as independent variables.

To evaluate the association between FEV_1_ at follow-up and FEV_1_ decline, respectively, and respiratory symptoms, two linear regression models were constructed (one for each dependent variable). Both models included data at study entry on recurrent wheeze, dyspnoea, longstanding cough and productive cough, and follow-up data on age and ICS use, BMI category at study entry, high BMI increase during follow-up and smoking habits from study entry to follow-up, and follow-up data on occupational GDF exposure as independent variables. Further, in attempt to adjust for potential bias due to regression to the mean, also FEV_1_pp at study entry was included as independent variable in the model with FEV_1_ decline as dependent variable.

These three models were performed separately for men and women, and for those with and without allergic rhinitis at study entry. To account for potential cohort bias, all models included adjustments for the original population cohorts (cohorts I–V).

Additional models were constructed by replacing the four included respiratory symptoms by one symptom at the time, by the number of symptoms, included as a categorical and a continuous variable respectively in separate models. Finally, additional models were constructed without including any type or number of respiratory symptoms. All models had R square values of 0.131–0.235, adjusted R square values of 0.099–0.199, ANOVA p<0.001, VIF <3 for all independent variables in the models (VIF <1.5 for the four included respiratory symptoms), and no signs of heteroscedasticity.

## Results

### Clinical characteristics

At study entry (1986–2001), the mean age was 40 years, the mean BMI 25.6, and the proportion of women was 55.7%. Overall, 44.5% were non-smokers, 28.4% were ex-smokers, 27.1% were current smokers, 34.7% had allergic rhinitis and 11.9% used ICS. Dyspnoea and longstanding cough were more common in women than men, and in both sexes the most frequent respiratory symptom was recurrent wheeze (table 1). Among women and men 91.6% and 86.6%, respectively, reported at least one of the four symptoms. At follow-up (2012–2014), the mean BMI change was 2.9 BMI units, 43.9% used ICS and 38% reported occupational exposure to GDF (online supplemental table 1).

10.1136/bmjresp-2021-000981.supp1 Supplementary data

**Table 1:** Characteristics and prevalence of respiratory symptoms at study entry among women, men and all participants

Characteristics	Women n=545	Men n=432	P value	All n=977
Age Mean (SD) in years	40.3 (12.0)	40.7 (11.4)	0.600	40.4 (11.7)
BMI mean (SD)	25.2 (4.5)	26.1 (3.4)	<0.001	25.6 (4.1)
BMI category			<0.001	
Underweight (BMI <20)	42 (7.7)	8 (1.9)		50 (5.1)
Normal (20≤BMI<25)	266 (48.8)	175 (40.5)		441 (45.1)
Overweight (25≤BMI<30)	145 (26.6)	183 (42.4)		328 (33.6)
Obese (BMI≥30)	78 (14.3)	55 (12.7)		133 (13.6)
BMI missing	14 (2.6)	11 (2.5)		25 (2.6)
Smoking habits			0.080	
Non-smoker	250 (45.9)	185 (42.8)		435 (44.5)
Ex-smoker	139 (25.5)	138 (31.9)		277 (28.4)
Current smoker	156 (28.6)	109 (25.2)		265 (27.1)
Any asthma medication use last 12 months	189 (34.7)	170 (39.4)	0.132	359 (36.7)
Inhaled corticosteroids use last 12 months			0.077	
As needed	13 (2.4)	22 (5.1)		35 (3.6)
Most days of the week	46 (8.4)	35 (8.1)		81 (8.3)
Allergic rhinitis	192 (35.2)	147 (34)	0.695	339 (34.7)
Cohort			0.054	
I	92 (16.9)	79 (18.3)		171 (17.5)
II	220 (40.4)	194 (44.9)		414 (42.4)
III	46 (8.4)	37 (8.6)		83 (8.5)
IV	56 (10.3)	52 (12)		108 (11.1)
V	131 (24)	70 (16.2)		201 (20.6)
Recurrent wheeze	430 (78.9)	332 (76.9)	0.443	762 (78.0)
Dyspnoea	175 (32.1)	64 (14.8)	<0.001	239 (24.5)
Longstanding cough	275 (50.5)	168 (38.9)	<0.001	443 (45.3)
Productive cough	195 (35.8)	151 (35.0)	0.789	346 (35.4)
Number of symptoms			<0.001	
None or one	206 (37.8)	216 (50.0)		422 (43.2)
Two	157 (28.8)	110 (25.5)		267 (27.3)
Three	127 (23.3)	87 (20.1)		214 (21.9)
Four	55 (10.1)	19 (4.4)		74 (7.6)

Basic characteristics presented as mean (SD) with p values from t-test and n (%) with p values from χ^2^ test.

The number of symptom groups include recurrent wheeze, dyspnoea, longstanding cough and productive cough.

### Type of respiratory symptoms at study entry in association with FEV_1_

Overall, men had lower mean FEV_1_pp than women (86.6 (SD 14.8) compared with 90.0 (SD 12.6), p<0.001) at study entry. Recurrent wheeze and dyspnoea were associated with lower FEV_1_pp both at study entry and follow-up, while longstanding cough was not associated with either of the two and productive cough was associated only with lower FEV_1_pp at follow-up. At study entry, the largest discrepancy in FEV_1_pp was found between subjects with and without dyspnoea (mean difference 4.9pp (p<0.001) in women and 5.7pp (p=0.005) in men) while at follow-up, it was between subjects with and without recurrent wheeze (mean difference 4.3pp (p=0.009) in women and 4.7pp (p=0.010) in men). Regarding the annual decline in FEV_1_, none of the symptoms associated significantly in either sex (table 2).

**Table 2:** FEV_1_ at study entry, follow-up, and annual decline, respectively, by type of respiratory symptoms among women and men

Symptom at study entry	FEV_1_pp pre-BD at study entry	FEV_1_pp pre-BD at follow-up	Annual decline in FEV_1_pp
Women			
Recurrent wheeze			
No (n=115)	92.6 (11.6)	93.7 (14.7)	0.087 (0.602)
Yes (n=430)	89.3 (12.8)	89.4 (15.6)	0.014 (0.608)
P value	**0.013**	**0.009**	0.254
Dyspnoea			
No (n=370)	91.6 (12.2)	91.5 (15.5)	0.006 (0.623)
Yes (n=175)	86.7 (12.9)	87.9 (15.4)	0.078 (0.570)
P value	**<0.001**	**0.012**	0.194
Longstanding cough			
No (n=270)	90.5 (12.9)	91.0 (16.0)	0.042 (0.573)
Yes (n=275)	89.5 (12.4)	89.7 (15.0)	0.017 (0.638)
P value	0.358	0.340	0.637
Productive cough			
No (n=350)	90.8 (12.1)	91.5 (14.8)	0.049 (0.589)
Yes (n=195)	88.6 (13.3)	88.2 (16.6)	−0.007 (0.638)
P value	0.051	**0.016**	0.303
Men			
Recurrent wheeze			
No (n=100)	90.1 (12.4)	86.5 (13.6)	−0.147 (0.576)
Yes (n=332)	85.5 (15.3)	81.8 (16.8)	−0.197 (0.638)
P value	**0.007**	**0.010**	0.482
Dyspnoea			
No (n=368)	87.4 (14.5)	83.5 (15.7)	−0.193 (0.613)
Yes (n=64)	81.7 (16.0)	79.2 (18.8)	−0.144 (0.685)
P value	**0.005**	**0.049**	0.563
Longstanding cough			
No (n=264)	87.3 (14.7)	84.0 (15.6)	−0.159 (0.588)
Yes (n=168)	85.4 (14.9)	81.2 (17.1)	−0.227 (0.675)
P value	0.192	0.086	0.275
Productive cough			
No (n=281)	87.5 (14.5)	84.0 (15.1)	−0.169 (0.624)
Yes (n=151)	84.8 (15.2)	80.7 (18.0)	−0.216 (0.625)
P value	0.063	**0.044**	0.463
Total			
Recurrent wheeze			
No (n=215)	91.4 (12.1)	90.3 (14.6)	−0.022 (0.600)
Yes (n=762)	87.7 (14.1)	86.1 (16.6)	−0.078 (0.629)
P value	**<0.001**	**0.001**	0.244
Dyspnoea			
No (n=738)	89.5 (13.5)	87.5 (16.1)	−0.093 (0.626)
Yes (n=239)	85.4 (14.0)	85.6 (16.8)	0.019 (0.609)
P value	**<0.001**	0.107	**0.016**
Longstanding cough			
No (n=534)	88.9 (13.9)	87.5 (16.2)	−0.058 (0.589)
Yes (n=443)	88.0 (13.5)	86.5 (16.3)	−0.075 (0.663)
P value	0.272	0.326	0.664
Productive cough			
No (n=631)	89.3 (13.4)	88.2 (15.4)	−0.048 (0.614)
Yes (n=346)	86.9 (14.3)	84.9 (17.6)	−0.098 (0.639)
P value	**0.008**	**0.003**	0.235

Results presented as Mean (SD) and p values from t-test, presented in bold when p<0.05

Pre-BD = pre-bronchodilatation

FEV_1_, forced expiratory volume in 1 s; Pre-BD, pre-bronchodilatation.

**Figure 1:**
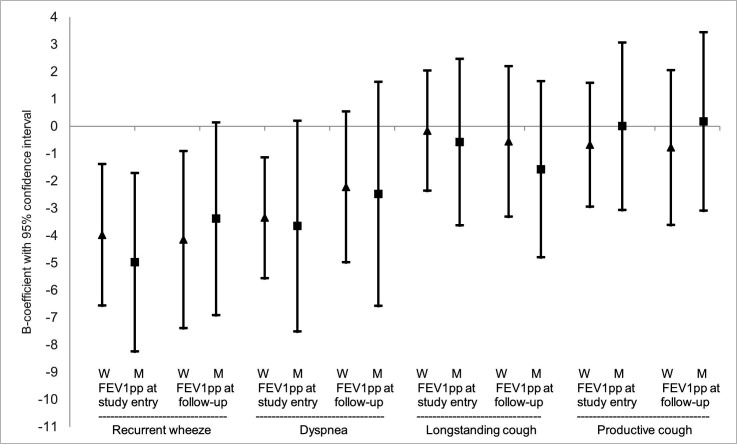
Association of type of respiratory symptoms at study entry with FEV_1_pp at study entry and followup after adjusting for other factors among women and men. Results expressed as B-coefficients with 95%confidence intervals from linear regression models, illustrating the mean difference in FEV_1_pp among those with vs without the symptom. The model with FEV_1_pp pre-BD at study entry as dependent variable included recurrent wheeze, dyspnea, longstanding cough, productive cough, age, BMI categories, smoking and original cohort as independent variables. The model with FEV_1_pp pre-BD at follow-up as dependent variable included recurrent wheeze, dyspnea, longstanding cough, productive cough, age, BMI categories, high BMI increase, smoking, ICS use, occupational exposure to GDF and original cohort as independent variables. W=Women (▲). M=Men (■).

In the adjusted regression models, recurrent wheeze remained associated with lower FEV_1_pp at study entry among both women and men, while at follow-up it remained significant only among women. Dyspnoea remained associated with lower FEV_1_pp at study entry among women (figure 1). None of the respiratory symptoms were associated with the annual decline in FEV_1_. The same adjusted model as above was reiterated, this time stratifying by those with and without allergic rhinitis (online supplemental figure 1), and once again recurrent wheeze had the strongest associations with lower FEV_1_pp at study entry and follow-up regardless of having allergic rhinitis or not.

### Number of respiratory symptoms at study entry in association with FEV_1_

A linear trend (the more symptoms, the lower FEV_1_pp) was seen both at study entry and at follow-up in both sexes (table 3). Among men, but not women, there was a significant association between the number of respiratory symptoms and annual decline in FEV_1_.

**Table 3:** FEV_1_ at study entry, follow-up and annual decline by number of respiratory symptoms among women, men and all participants

		FEV_1_pp pre-BD at study entry	FEV_1_pp pre-BD at follow-up	Annual decline in FEV_1_pp
Women				
None or 1 symptoms	n=206	92.3 (11.8)	92.8 (15.1)	0.040 (0.580)
2 symptoms	n=157	89.5 (12.7)	90.2 (15.2)	0.048 (0.611)
3 symptoms	n=127	88.8 (12.8)	87.9 (16.0)	−0.035 (0.666)
4 symptoms	n=55	85.5 (13.4)	87.0 (15.8)	0.084 (0.550)
P value		**0.001**	**0.011**	0.553
Men				
None or 1 symptoms	n=216	89.2 (14.1)	85.0 (14.8)	−0.207 (0.583)
2 symptoms	n=110	84.6 (14.4)	83.2 (15.3)	−0.044 (0.670)
3 symptoms	n=87	83.1 (16.1)	78.0 (19.0)	−0.287 (0.606)
4 symptoms	n=19	83.3 (15.0)	79.0 (19.3)	−0.303 (0.772)
P value		**0.002**	**0.005**	**0.031**
Total				
None or 1 symptoms	n=422	90.7 (13.1)	88.8 (15.4)	−0.086 (0.594)
2 symptoms	n=267	87.5 (13.6)	87.3 (15.6)	0.010 (0.636)
3 symptoms	n=214	86.5 (14.5)	83.8 (17.9)	−0.137 (0.652)
4 symptoms	n=74	84.9 (13.7)	84.9 (17.0)	−0.016 (0.632)
P value		**<0.001**	**0.002**	0.052

Results presented as mean (SD) and p values from ANOVA in bold figures indicate p<0.05.

Included respiratory symptoms: recurrent wheeze, dyspnoea, longstanding cough, productive cough.

ANOVA, analysis of variance; FEV_1_, forced expiratory volume in 1 s; Pre-BD, pre-bronchodilatation.

**Figure 2:**
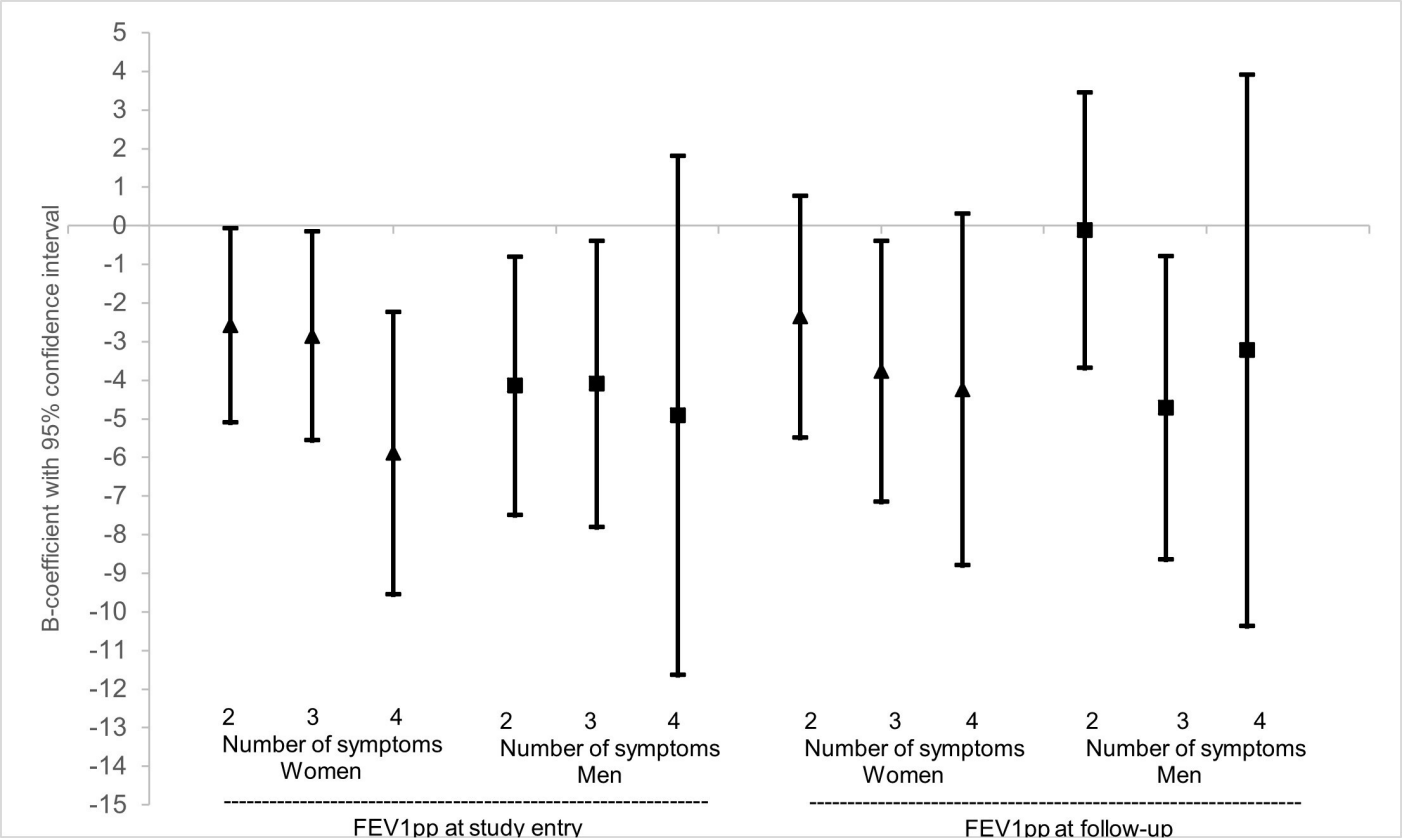
Association of number of respiratory symptoms at study entry with FEV_1_pp at study entry and follow-up after adjusting for other factors among women and men. Results expressed as B-coefficients with 95% confidence intervals from linear regression models, illustrating the mean difference in FEV_1_pp among those with 2, 3 or 4 symptoms vs those with 0-1 symptom. The model with FEV_1_pp pre-BD at study entry as dependent variable included the number of respiratory symptoms, age, BMI categories, smoking and original cohort as independent variables. Having 0-1 symptoms was used as reference category. The model with FEV_1_pp pre-BD at follow-up as dependent variable included the number of respiratory symptoms age, BMI categories, high BMI increase, smoking, ICS use, occupational exposure to GDF and original cohort as independent variables. Having 0-1 symptoms was used as reference category. W=Women (▲). M=Men (■).

In the adjusted regression models, women having 2, 3 or 4 symptoms had significantly lower FEV_1_pp at study entry than those with 0–1 symptom, and a similar trend was observed at follow-up. Among men, having 2 or 3 symptoms were significantly associated with lower FEV_1_pp at study entry, while the trend was less clear at follow-up (figure 2). When the number of symptoms was added to the models as a continuous variable, significant associations were seen both at study entry and follow-up for both sexes (online supplemental figure 2).

### Non-participant analyses of the type and number of respiratory symptoms in association with FEV_1_ at study entry

A non-participant analysis was performed to estimate potential selection bias. It revealed lower FEV_1_pp compared with participants and confirmed that recurrent wheeze, dyspnoea, and productive cough associated with lower FEV_1_pp at study entry. Regarding the number of respiratory symptoms, this analysis showed an even more accentuated linear trend by number of symptoms in both sexes (online supplemental table 2).

### Other factors in association with FEV_1_ at study entry, follow-up, and annual decline

For all three FEV_1_ outcomes, the strongest negative associations were found for current smoking. Also, those that stopped smoking in between study entry and follow-up (quitters) had lower FEV_1_pp at follow-up and a more rapid annual decline, while ex-smokers had not. Male sex also associated with lower FEV_1_pp at study entry and follow-up and annual decline. Being obese at study entry was significantly associated with lower FEV_1_pp at study entry and annual decline, but not at follow-up. However, high BMI increase from entry to follow-up (>4.92 BMI units) was significantly associated with lower FEV_1_ both at follow-up and with a more rapid annual decline in FEV_1_ (online supplemental table 3).

## Discussion

In this population-based prospective study of an adult asthma cohort, recurrent wheeze and dyspnoea associated with lower FEV_1_ at study entry, among both women and men regardless of having allergic rhinitis or not. After on average 18 years follow-up time, this association prevailed for recurrent wheeze and dyspnoea, and appeared for productive cough. Further, the higher number of respiratory symptoms at study entry, the lower the FEV_1_ both at study entry and follow-up. Also, in the long-term perspective, recurrent wheeze was a predictor of low FEV_1_ independently of ICS, smoking habits, BMI, and other potentially confounding factors, in contrast to dyspnoea and productive cough. Longstanding cough was neither associated with FEV_1_ at study entry nor at follow-up. None of the respiratory symptoms associated with an unfavourable rate of FEV_1_ decline, instead the results showed that the lower FEV_1_ mainly was manifested already at study entry and that FEV_1_ remained at a similarly low level also at the long-term follow-up.

To our knowledge, there are no previous prospective asthma cohort studies on associations between respiratory symptoms and changes in FEV_1_. Some cross-sectional^16 25^ and prospective^14 15^ studies of population samples have been performed previously, of which most have shown that both smoking and potentially harmful occupational exposures are more common among men, and that men tend to have lower lung function than women,^15 25 26^ in line with our results. Studies on chronic obstructive pulmonary disease (COPD) have however shown that respiratory symptoms (mainly chronic productive cough) predict both lower lung function levels and a more rapid lung function decline.^27 28^

An interesting finding in our study was that participants without recurrent wheeze had the highest mean FEV_1_ both at study entry and at follow-up. After adjustment, having recurrent wheeze remained associated with lower FEV_1_ at study entry in both sexes. In addition, this association remained also with lower FEV_1_ at follow-up after adjustment, however only significant in women, implying that recurrent wheeze is able to predict remaining low lung function also over a long time period among women with asthma independently of age, BMI, smoking, occupational exposure to GDF, ICS use, and other respiratory symptoms. If ICS at follow-up had not been included in the model, the association between recurrent wheeze and lower FEV_1_ at follow-up would have remained significant also in men. A possible explanation would be confounding by indication where subjects using ICS may have a worse asthma profile and more impaired lung function,^29^ that is, ICS not being the cause of this impairment, as their use has been proven to decrease FEV_1_ decline^30^ and partially prevent asthma exacerbations which also are associated with a reduced lung function decline.^31^

Women with dyspnoea had the lowest mean FEV_1_ at study entry, but still preserved, that is, equally low, FEV_1_ at follow-up, possibly a consequence of several gender aspects.^32^ When exploring FEV_1_ in relation to having both recurrent wheeze and dyspnoea in a cross-sectional setting, this group had indeed the lowest FEV_1_ at study entry among all symptom combinations. The finding that women with dyspnoea at study entry had preserved instead of further decreased FEV_1_ values at follow-up could potentially be related to the fourfold increase in ICS use from study entry to follow-up. One partial explanation, besides a substantial general increase in prescriptions of ICS during the study period,^33^ is that our cohort includes both prevalent and incident asthmatics with and without treatment at study entry, and that many started treatment shortly after study entry.^18^

In contrast to recurrent wheeze, productive cough was more related to smoking, which in turn associated with lower FEV_1_. Similar results were obtained in a previous study from Northern Sweden where subjects with wheezing in combination with attacks of breathlessness had lower FEV_1_ than those with chronic productive cough.^25^ In that study, after adjusting for other factors including smoking habits, persistent wheeze yielded the strongest association with decreased lung function while smoking was strongly associated with chronic productive cough.^25^ Results from the European Community Respiratory Health Survey have shown that smoking may be more strongly associated with FEV_1_ decline among adults with than without asthma,^26^ and the parallel Tucson and Cracow studies have in turn shown that chronic cough may be an indicator of increased effect of smoking on FEV_1_, which is supported by our findings. Although Fletcher and Peto showed in the 1970s that chronic mucus hypersecretion did not associate with accelerated decline in FEV_1_ in a cohort of men followed for 8 years,^14^ our study showed that after following both women and men with asthma over a long time period, this association was indeed observed for productive cough, and it was related to smoking.

Asthma is a heterogenous disease, and one could speculate that both lung function^34^ and symptom profiles could differ between phenotypes. Such indications have been found in other studies, where for instance the obese asthma phenotype has been more commonly associated with wheeze^35^ and dyspnoea.^36^ Productive cough, on the other hand presents more often in smokers,^25^ and both smoking and accelerated lung function decline are related to the neutrophilic asthma phenotype as well as COPD.^29^ From a treatment perspective, prognosis may differ between asthma phenotypes, for example allergic and non-allergic asthma,^4^ why this distinction may be of particular interest. We explored this scenario by comparing among those with and without allergic rhinitis but could not find different symptom patterns with regards to associations to FEV_1_. Instead, recurrent wheeze was strongly associated with lower FEV_1_ regardless of having allergic rhinitis or not.

Regarding the number of symptoms at study entry, we found that the more symptoms, the lower the FEV_1_. Our non-participant analysis showed stronger associations between the number of symptoms and lower FEV_1_, along with markedly lower FEV_1_ level as previously shown^17^ among non-participants than participants. A Norwegian population-based prospective cohort study found increasing mortality by number of symptoms,^8^ and low FEV_1_ has also repeatedly been associated with mortality.^10 17^ The adjusted analyses on groups having at least 2, 3 or 4 symptoms respectively, that is, not treating the number of symptoms as a continuous variable, did not reveal a completely linear trend, especially among men, implying that each symptom may not have equal effect on FEV_1._ Instead, some specific symptoms may have a stronger effect, particularly recurrent wheeze.

Weaknesses of our study include the long time between examinations causing a potential healthy survivor bias. A previous study of this cohort assessing factors related to all-cause mortality and non-participation on follow-up concluded that deceased subjects were predominantly male, current smokers, with low socioeconomic status, ischaemic heart disease and lower FEV_1_. Regarding non-participants at follow-up, besides having more ischaemic heart disease and lower socioeconomic status they were also more frequently obese at study entry, compared with those participating at follow-up.^17^ A way to estimate potential selection bias of healthy subjects for our study was by performing the non-participant analysis, which showed stronger associations between symptoms and low lung function compared with among participants. Nevertheless, our study revealed important associations, but the magnitude of our findings is probably underestimated. Other weaknesses include the different time points at study entry, that treatment options and guidelines changed between 1986 and 2001, and the lack of information on asthma medication use in-between examinations. In attempt to account for this, all regression analyses were adjusted for the original cohorts. Analyses on FEV_1_ decline may be subject to bias due to regression to the mean, but this was addressed by including FEV_1_pp at study entry in these models. Different types of spirometers at study entry and follow-up is a potential weakness because this may alter the comparability of the lung function values between study entry and follow-up and thus affect estimates of FEV_1_ decline. However, as this was the case for all subjects, no systematic bias was introduced in the comparisons between those with and without different symptoms.

This study has several strong points such as the high participation and retention rate, strong statistical power and the real-life study design that may mirror the everyday reality. There are several factors known to be related to lung function decline among adults with asthma such as obesity,^37 38^ smoking habits^39 40^ and treatment,^31^ which are important to take into account to establish if respiratory symptoms are associated to lung function independently of these factors. The large sample and rich data of this cohort allowed for adjustment for multiple such important possibly confounding factors, where particularly smoking and obesity were identified as strongly associated with lower FEV_1_. Several different regression models were evaluated, and those including only one symptom at the time in the model confirmed the main findings of strongest associations especially with recurrent wheeze and also with dyspnoea (online supplemental table 4). The long follow-up time can also be a strength by enabling evaluation of long-term prognosis. Our study is unique due to the limited number of prospective studies of asthma cohorts addressing the association between symptoms and lung function over time, also having the sample size able to allow for stratification, which is valuable to illustrate these associations among women and men separately.

In conclusion, recurrent wheeze and dyspnoea at study entry associated with lower lung function both at study entry and follow-up among women and men with asthma. Importantly, recurrent wheeze remained a predictor for lower lung function in the long run also after adjusting for several potential confounders, especially among women. Also the number of symptoms can predict lower lung function in a long-term perspective.

## Data Availability

Data are available upon reasonable request.
