# Supplementary material for: Among respiratory symptoms, wheeze associates most strongly with impaired lung function in adults with asthma: a long-term prospective cohort study
Source: BMJ Open Respir Res. 2021 Jul 18;8(1):e000981. doi: 10.1136/bmjresp-2021-000981 (PMC8291305; doi:10.1136/bmjresp-2021-000981)
Supplement: Supplementary data [file bmjresp-2021-000981supp001.pdf]

SUPPLEMENTAL MATERIAL

Supplemental Table 1 Characteristics at follow-up among women, men and all participants

Characteristics	Women N=545	Men N=432	All N=977
Age Mean (SD) in years	58.7 (12.8)	59.4 (12.0)	59.0 (12.4)
BMI Mean (SD)	28.2 (5.5)	28.9 (4.5)	28.5 (5.1)
BMI group			
Underweight (BMI<20)	17 (3.1)	2 (0.5)	19 (1.9)
Normal (20<=BMI<25)	144 (26.4)	72 (16.7)	216 (22.1)
Overweight (25<=BMI<30)	211 (38.7)	207 (47.9)	418 (42.8)
Obese (BMI=>30)	173 (31.7)	150 (34.7)	323 (33.1)
BMI missing	0 (0)	1 (0.2)	1 (0.1)
BMI change Mean (SD)	3.0 (4.0)	2.8 (3.1)	2.9 (3.6)
High BMI increase (>4.92)	143 (26.2)	94 (21.2)	237 (24.2)
Smoking habits			
Non-smoker	268 (49.2)	199 (46.1)	467 (47.8)
Ex-smoker	207 (38.0)	189 (43.8)	396 (40.5)
Current smoker	70 (12.8)	44 (10.2)	114 (11.7)
Inhaled corticosteroid use	265 (48.6)	164 (38.0)	429 (43.9)
Work GDF exposure	112 (20.6)	259 (60.0)	371 (38.0)
ER visits last 12 months	50 (9.2)	25 (5.8)	75 (7.7)
Hospitalizations las 12 months	10 (1.8)	10 (2.3)	20 (2)
Oral corticosteroid use			
Maintenance treatment	2 (0.4)	1 (0.2)	3 (0.3)
Only with exacerbations	11 (2)	3 (0.7)	14 (1.4)

BMI missing at follow-up = 1 man

BMI change was calculated for 531 women and 420 men.

Bermúdez Barón N, *et al. BMJ Open Resp Res* 2022; 8:e000981. doi: 10.1136/bmjresp-2021-000981

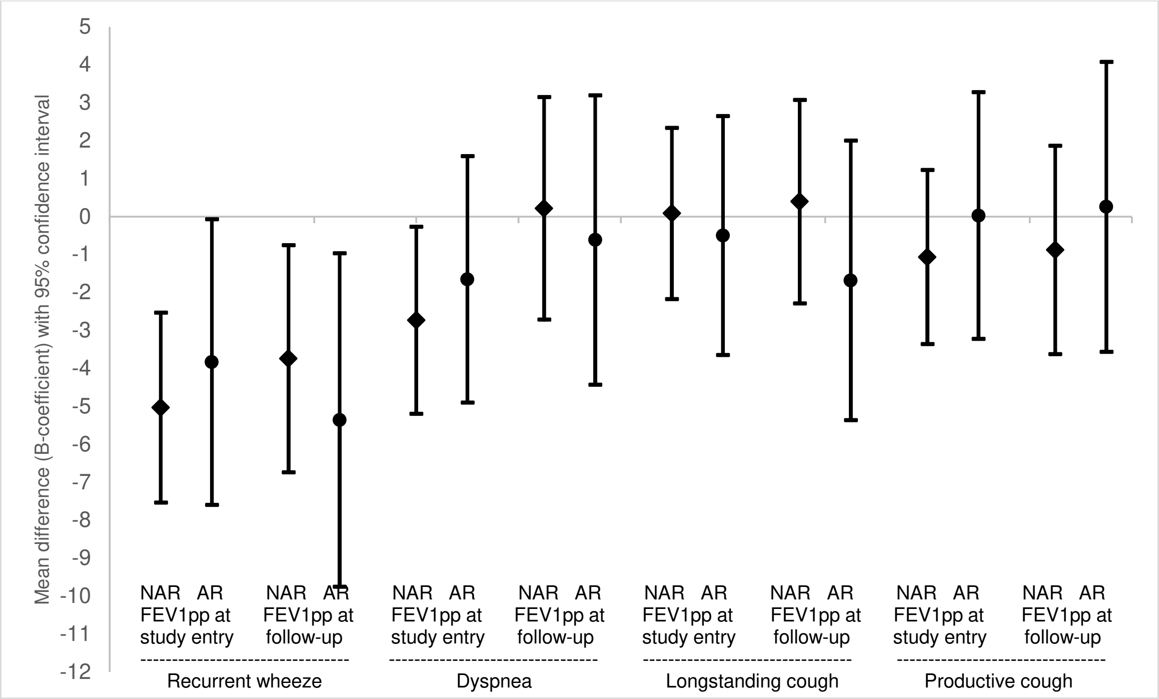

Supplemental Figure 1 Association of type of respiratory symptoms at study entry with FEV_1pp at study entry and follow-up after adjusting for other factors among those with and without allergic rhinitis. Results expressed as B-coefficients with 95% confidence intervals from linear regression models, illustrating the mean difference in FEV_1pp among those with vs without allergic rhinitis.

The model with FEV_1pp pre-BD at study entry as dependent variable included recurrent wheeze, dyspnea, longstanding cough, productive cough, age, BMI category, smoking and original cohort as independent variables.
The model with FEV_1pp pre-BD at follow-up as dependent variable included recurrent wheeze, dyspnea, longstanding cough, productive cough, age, BMI category, high BMI increase smoking, ICS use, occupational exposure to GDF and original cohort as independent variables.
NAR=No allergic rhinitis (♦). AR=Allergic rhinitis (●).

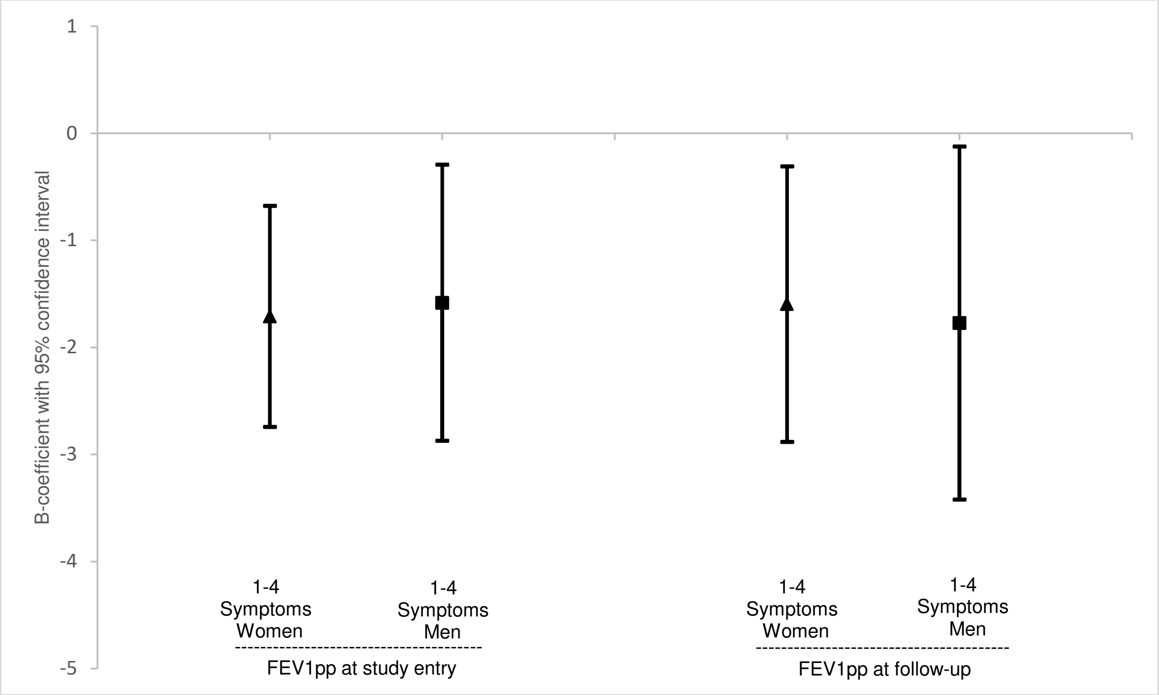

Supplemental Figure 2 Association of number of respiratory symptoms at study entry as a continuous variable with FEV_1pp at study entry and follow-up after adjusting for other factors among women and men. Results expressed as B-coefficients with 95% confidence intervals from linear regression models, illustrating the mean difference in FEV_1pp among those with 1 vs 2, 2 vs 3 and 3 vs 4 symptoms.

The model with FEV_1pp pre-BD at study entry as dependent variable included the number of respiratory symptoms, age, BMI category, smoking and original cohort as independent variables.
The model with follow-up FEV_1pp pre-BD as dependent variable included the number of respiratory symptoms, age, BMI category, high BMI increase, smoking, ICS use, occupational exposure to GDF and original cohort as independent variables.
W=Women (▲). M=Men (■). Those reporting 0 symptoms were merged with those reporting 1 symptom in this analysis.

Supplemental Table 2 FEV_1pp pre-BD at study entry by type and number of respiratory symptoms among non-participants at follow-up

Women N=574			Men N=478			All N=1052		
Recurrent wheeze	No	82.7 (21.0)	Recurrent wheeze	No	81.8 (20.3)	Recurrent wheeze	No	82.3 (20.7)
	Yes	79.5 (20.3)		Yes	76.4 (18.7)		Yes	78.1 (19.7)
	P-value	0.119		P-value	**0.012**		P-value	**0.004**
Dyspnea	No	85.1 (17.3)	Dyspnea	No	82.9 (16.4)	Dyspnea	No	84.0 (16.9)
	Yes	73.6 (22.7)		Yes	66.5 (19.8)		Yes	70.8 (21.8)
	P-value	**<0.001**		P-value	**<0.001**		P-value	**<0.001**
Longstanding cough	No	80.7 (20.2)	Longstanding cough	No	78.6 (18.5)	Longstanding cough	No	79.7 (19.4)
	Yes	79.8 (20.9)		Yes	76.2 (19.9)		Yes	78.2 (20.5)
	P-value	0.591		P-value	0.174		P-value	0.224
Productive cough	No	83.0 (18.5)	Productive cough	No	83.6 (16.1)	Productive cough	No	83.3 (17.5)
	Yes	77.1 (22.2)		Yes	72.1 (20.1)		Yes	74.7 (21.3)
	P-value	**0.001**		P-value	**<0.001**		P-value	**<0.001**
0-1 symptoms		85.8 (17.0)	0-1 symptoms		84.2 (16.9)	0-1 symptoms		85.0 (16.9)
2 symptoms		79.8 (21.0)	2 symptoms		77.3 (17.0)	2 symptoms		78.7 (19.3)
3 symptoms		78.8 (20.3)	3 symptoms		76.2 (18.6)	3 symptoms		77.6 (19.6)
4 symptoms		71.9 (23.4)	4 symptoms		64.8 (21.5)	4 symptoms		68.7 (22.8)
P-value		**<0.001**	P-value		**<0.001**	P-value		**<0.001**

Results for the type of symptoms presented as n (%) and p-values from T-test in bold figures indicate p<0.05

Results for the number of symptoms presented as Mean (Standard deviation) and p-values from ANOVA in bold figures indicate p<0.05

pre-BD = pre-bronchodilatation. Non-participants at follow-up (n=1078). Missing FEV1pp at study entry (n=26)

Supplemental Table 3 Analysis of potential confounders by linear regression with results expressed as B-coefficients with corresponding 95% confidence intervals and p-values

	Total N=977		
	**B**	**95% Confidence Interval**	**P-value**
**FEV1pp pre-BD at study entry**			
Sex (Male)	-3.55	(-5.22 - -1.88)	**<0.001**
Age at study entry	-0.32	(-0.39 - -0.24)	**<0.001**
Underweight	-2.95	(-6.76 - 0.86)	0.129
Overweight	-0.78	(-2.69 - 1.13)	0.423
Obese	-3.34	(-5.88 - -0.79)	**0.010**
BMI missing	-2.30	(-7.63 - 3.02)	0.397
Ex-smokers at study entry	-0.83	(-2.82 - 1.16)	0.412
Current smokers at study entry	-2.96	(-4.95 - -0.97)	**0.004**
**FEV1pp pre-BD at follow-up**			
Sex (Male)	-8.78	(-10.88 - -6.68)	**<0.001**
Age at follow-up	-0.27	(-0.36 - -0.19)	**<0.001**
Underweight at study entry	-4.47	(-8.84 - -0.11)	0.045
Overweight at study entry	-0.04	(-2.23 - 2.14)	0.968
Obese at study entry	-0.73	(-3.64 - 2.19	0.624
BMI missing at study entry	0.55	(-5.46 - 6.57)	0.857
High BMI increase	-2.89	(-5.13 - -0.65)	**0.012**
Ex-smokers at follow-up	-1.68	(-4.03 - 0.68)	0.162
Quitters at follow-up	-7.40	(-10.24 - -4.56)	**<0.001**
Current smokers at follow-up	-12.44	(-15.54 - -9.33)	**<0.001**
ICS use at follow-up	-5.93	(-7.86 - -4.00)	**<0.001**
Occupational GDF exposure at follow-up	0.66	(-1.44 - 2.76)	0.539
**Annual decline in FEV1pp**			
Sex (Male)	-0.23	(-0.31 - -0.14)	**<0.001**
Age at follow-up	0.00	(0.00 - 0.00)	0.485
Underweight at study entry	-0.10	(-0.28 - 0.08)	0.266
Overweight at study entry	0.06	(-0.03 - 0.15)	0.197
Obese at study entry	0.13	(0.02 - 0.25)	**0.027**
BMI missing at study entry	0.05	(-0.19 - 0.30)	0.667
High BMI increase	-0.16	(-0.25 - -0.07)	**<0.001**
Ex-smokers at follow-up	-0.06	(-0.16 - 0.03)	0.197
Quitters at follow-up	-0.16	(-0.28 - -0.05)	**0.005**
Current smokers at follow-up	-0.37	(-0.50 - -0.25)	**<0.001**
ICS use at follow-up	-0.03	(-0.11 - 0.05)	0.457
Occupational GDF exposure at follow-up	-0.03	(-0.11 - 0.06)	0.519

B = Beta-coefficient from linear regression models. Bold values indicate p<0.05

Normal weight at study entry (reference category) (n=441)

Underweight (n=50), Overweight (n=328), Obese (n=133), BMI missing (n=75) (not shown)

BMI increase < 4.92 (reference category) (n=714), BMI increase > 4.92 (n=237)

Never smokers at study entry (reference category) (n=435)

Ex-smokers (n=277), Current Smokers (n=265)

Cohort I (reference category) (n=171)

Cohort II (n=414), Cohort III (n=83), Cohort IV (n=108), Cohort V (n=201). Results not shown.

Constantly non-smokers (reference category) (n=418)

Ex-Smokers at follow-up = Non-smokers or ex-smokers to ex-smokers (n=253)

Quitters = Smokers to ex-smokers (n=143)

Current smokers at follow-up = Non, ex, or current smokers to smokers (n=114)

Follow-up smoking residual group (n=49) (not shown)

ICS use at follow-up = Yes (n=429) No (n=548)

Occupational GDF exposure at follow-up = Yes (n=371) No (n=606)

Cohort I (reference category) (n=171)

Cohort II (n=414) Cohort III (n=83) Cohort IV (n=108) Cohort V (n=201). Results not shown

Supplemental Table 4 Regression analyses for FEV1pp at study entry and follow-up and each type of symptom both unadjusted and adjusted with results expressed as B-coefficients with corresponding 95% confidence intervals

		Unadjusted		Adjusted	
		B	95% Confidence interval	B	95% Confidence interval
**FEV1pp pre-BD at study entry**					
Recurrent wheeze	Women	-3.30	(-5.88 - -0.71)	-4.49	(-7.08 - -1.91)
	Men	-4.55	(-7.85 - -1.25)	-5.38	(-8.61 - -2.15)
Dyspnea	Women	-4.82	(-7.06 - -2.59)	-3.84	(-6.03 - -1.65)
	Men	-5.68	(-9.59 - -1.77)	-4.35	(-8.17 - -0.53)
Longstanding cough	Women	-1.00	(-3.12 - 1.13)	-0.85	(-2.93 - 1.23)
	Men	-1.91	(-4.78 - 0.96)	-1.05	(-3.96 - 1.86)
Productive cough	Women	-2.20	(-4.41 - 0.01)	-1.28	(-3.41 - 0.86)
	Men	-2.78	(-5.71 - 0.15)	-0.99	(-3.89 - 1.92)
**FEV1pp pre-BD at follow-up**					
Recurrent wheeze	Women	-4.24	(-7.42 - -1.05)	-4.50	(-7.72 - -1.28)
	Men	-4.73	(-8.35 - -1.12)	-3.74	(-7.23 - -0.26)
Dyspnea	Women	-3.59	(-6.37 - -0.80)	-2.77	(-5.50 - -0.04)
	Men	-4.33	(-8.64 - -0.02)	-2.97	(-7.02 - 1.07)
Longstanding cough	Women	-1.27	(-3.89 - 1.34)	-1.14	(-3.71 - 1.44)
	Men	-2.75	(-5.89 - 0.39)	-1.83	(-4.89 - 1.24)
Productive cough	Women	-3.33	(-6.04 - -0.61)	-1.38	(-4.03 - 1.28)
	Men	-3.30	(-6.50 - -0.09)	-0.82	(-3.90 - 2.26)

Unadjusted models include the respective symptom only

Unadjusted models had R square values of 0.002-0.032 and adjusted R square values of 0.001-0.030.

Adjusted models with FEV1pp pre-BD at study entry include the respective symptom, age BMI categories, smoking and original cohort as independent variables

Adjusted models with FEV1pp pre-BD at follow-up include the respective symptom, age BMI categories, high BMI increase, smoking, ICS use, occupational exposure to GDF and original cohort as independent variables

Adjusted models had R square values of 0.127-0.230 and adjusted R square values 0.103-0.199.

Bermúdez Barón N, *et al. BMJ Open Resp Res* 2022; 8:e000981. doi: 10.1136/bmjresp-2021-000981
